# Variability in tibia-fibular geometry is associated with increased tibial strain from running loads

**DOI:** 10.1098/rsos.230262

**Published:** 2023-09-27

**Authors:** Meghan Keast, Jason Bonacci, Aaron Fox

**Affiliations:** School of Exercise and Nutrition Sciences, Deakin University, 75 Pigdons Road, Waurn Ponds, 3216 Victoria, Australia

**Keywords:** statistical shape model, finite-element model, lower extremity, bone model, skeletal geometry

## Abstract

Variation in tibial geometry may alter strain magnitude and distribution during locomotion. We investigated the effect of tibia-fibula geometric variations on tibial strain with running loads applied at various speeds. Participant-specific three-dimensional models of the tibia-fibula were created using lower limb computed tomography scans from 30 cadavers. Finite-element models were developed in FEBio, and running loads from 3, 4 and 5 m s^−1^ were applied to extract effective strain from the tibial shaft. Linear regression models evaluated the relationship between geometric characteristics and effective strain along the tibial shaft. We found a statistically significant positive relationship between: (i) increased thickness of the midshaft to upper tibia with increased condyle prominence and effective strain at points along the distal anterolateral and proximal posterior regions of the tibial shaft; and (ii) increased midshaft cortical thickness and effective strain at points along the medial aspect of the distal tibial shaft. It is possible that increased thickness in the more proximal region of the tibia causes strain to redistribute to areas that are more susceptible to the applied loads. A thickness imbalance between the upper and distal portions of the tibial shaft could have a negative impact on tibial stress injury risk.

## 1. Introduction

Tibial stress injuries are one of the most common injuries in individuals that perform high volumes of running [1], and account for approximately 1 in 10 running-related injuries [2]. Tibial stress injuries are multifactorial in nature, but the most cited cause is mechanical fatigue resulting in microcrack accumulation degrading the material properties of the tibial bone [3–5]. The magnitude of strain applied to the tibia plays a key role in the development of this mechanical fatigue [6]. Tibial stress injury risk is influenced by several intrinsic and extrinsic factors, one of these being skeletal geometry [7]. Tibial geometry could influence the magnitude of strain on the bone and potential tibial stress injury risk. Previous studies have linked tibial geometric characteristics and tibial stress injury risk. These include geometries such as smaller tibias [8,9], thinner mid-diaphysis [7] and decreased cortical thickness [8,10]. These studies do not investigate the underpinning mechanisms of how these variations impact tibial strain magnitude and distribution at the bone level. Further, it is unclear if there are additional geometric variations of the tibia and fibula that could impact strain magnitude and potential tibial stress injury risk. Improved knowledge of how tibial geometric variation affects strain during running could advance our understanding of why specific geometries increase the risk of tibial stress injuries.

Previous literature has identified several geometric variations of the tibia and fibula that could impact tibial strain magnitude [11–13]. Variations include general tibia-fibula size; overall and midshaft thickness; prominence and size of the condyle plateau, tibial tuberosity and anterior crest; axial torsion of the tibial shaft; medulla cavity diameter; cortical thickness; anterior–posterior shaft curvature of both the cortical and trabecular; and the volume of trabecular bone in the proximal and distal ends of the tibia. However, to our knowledge, research analysing the effects of tibia-fibula shape on tibia strain during running is limited to one study [14]. Geometric variations of decreased tibial size, shorter tibia-fibula and increased sagittal plane curvature of the tibia were found to be associated with increases in strain magnitude when modelled in young active adults [14]. This modelling involved the isolated manipulation of shape and density components of a statistical appearance model to investigate changes in tibial strain measures related to specific characteristics [14]. Bone size and width, tibial curvature and cortical thickness were identified as having the largest impact on tibial bone strain [14]. This approach of altering appearance model components allows an understanding of how isolated shape and/or density characteristics modify tibial strain. However, the examined models represent a ‘synthetic’ tibia-fibula where concurrent shape variation is not considered. Estimating strain using participant-specific bone models, and finite-element analysis in conjunction with principal component analysis provides the ability to examine the effects of geometric variations on strain in realistic skeletal geometry.

The purpose of this study is to investigate the effect of tibia-fibula variation on tibial strain magnitude with running loads at various speeds applied using participant-specific models. It is hypothesized that, of previously identified geometric variations, decreases in overall tibia width and length will be associated with increases in tibial strain across the entire shaft of the tibia with increases in running speed. Further, increases in cortical thickness will be associated with a decrease in effective strain across the tibial shaft. Lastly, increased anterior–posterior bend of the tibial shaft will be associated with increased tibial strain along the mid-distal portion of the tibial shaft.

## 2. Methods

### 2.1. Finite-element analysis

#### 2.1.1. Models

This research was conducted using a shared dataset from an existing paper by the research team. Full details regarding the segmentation processes and shape model characteristics can be found in the previous work [12].

In summary, participant-specific three-dimensional models of the tibia-fibula were created using lower limb computed tomography (CT) scans from the right tibia and fibula of 30 cadavers (male *n* = 20, female *n* = 10) in the New Mexico Decedent Image Database (NMDID) [15]. The images were collected using a Phillips Brilliance Big Bore, with an acquisition setting of 120 kilovoltage peak (kVp) and 200 milliampere-seconds (mA s) with an in-plane resolution of 512 × 512 pixels and a slice thickness of 0.5 mm. Individuals whose records indicated participation in impact-based physical activities throughout life were selected for inclusion (i.e. team sports, dancing, recreational running and walking). This criterion was included to ensure participants were sampled from a generally active population. Included individuals had a mean (± s.d.) age of 28.7 ± 6.7 years, living body mass of 70.22 ± 11.36 kg and living height of 176.06 ± 11.61 cm. The tibias and fibulas were segmented from the CT images using Mimics innovation suite (Materialise, Leuven, Belgium). Tibias were segmented into two surfaces representing the outer boundaries of the trabecular and cortical bone, while fibulas were segmented as one surface representing the outer shape of the entire bone. Cortical bone was segmented using a predefined threshold (lower bound 700 Hounsfield units (HU), upper bound 2500 HU), all surfaces were checked, and manual corrections were applied where necessary. The inner border of the cortical bone was used to specify the beginning of the trabecular bone, all area inside this was segmented as the trabecular bone, including the medullary cavity. The resolution of the CT scans used in this study was not high enough to allow intricate segmentation of the lattice structure of the trabecular bone.

The segmented tibia-fibula surfaces were converted to volumetric meshes using quadratic tetrahedral elements using GIBBON toolbox [16], which uses TetGen software [17]. The tibia and fibula meshes contained a minimum of 246 641 and 36 482 elements, respectively, in line with mesh densities of previous work [14]. Bone was modelled as an orthotropic elastic material [14,18], with differing properties assigned for cortical and trabecular regions of the tibia, with the entire fibula volume considered as a cortical region. We used a generic elastic modulus of bone in the axial direction (i.e. *E*_3_), with 18.6 and 10.4 GPa allocated for cortical and trabecular regions, respectively [19]. We made the choice to use generic densities for the cortical and trabecular regions within the finite element (FE) models to remove any effect of bone density variation on our results—and subsequently isolate the effect of shape characteristics. The remaining constants for the orthotropic elastic material were obtained assuming constant anisotropy, where *E*_1_ = 0.574 · *E*_3_, *E*_2_ = 0.577 · *E*_3_, *G*_12_ = 0.195 · *E*_3_, *G*_23_ = 0.265 · *E*_3_, *G*_3_1 = 0.216 · *E*_3_, *ν*_12_ = 0.427, *ν*_23_ = 0.234 and *ν*_31_ = 0.405 (subscripts 1–3 refer to the medial–lateral, anterior–posterior and axial directions, respectively) [14,18,20].

#### 2.1.2. Boundary constraints

Boundary constraints within FE simulations were similar to previous work [14,18]. Surface nodes at the tibial plateau were identified at the proximal end of the tibia [21] and constrained by fixing these nodes in translation and rotation. The closest surface node to the medial malleoli was constrained in the anterior–posterior direction. We used spring elements and tied-elastic contact to constrain the motion of the fibula relative to the tibia at the proximal and distal ends [18]. The stiffness of the ligament springs was set at 133, 166, 78 and 101 N mm^−1^ for the proximal anterior, proximal posterior, distal anterior and distal proximal ligaments, respectively [18,22,23].

#### 2.1.3. Loading conditions

We used an existing study of human running at 3, 4 and 5 m s^−1^ to provide the loads for our FE simulations [24]. OpenSim 4.3 [25] was used to extract ankle joint contact forces, and lower limb muscle forces and their lines of action from 10 male long-distance runners (29 ± 5 years; 70.9 ± 7.0 kg; 1.77 ± 0.04 m). Muscle forces and lines of action were extracted in the tibial coordinate system using a custom OpenSim plugin [26]. The average forces from the dataset (normalized to body mass) were extracted and used as the generic running loads within our FE simulations.

The average ankle joint contact and muscle forces at the time of peak resultant ankle joint contact force were scaled to each participant's living body mass. The closest surface node to the ankle joint centre was identified [21] and the ankle joint contact force applied as a concentrated nodal force to this point. Insertion points of 16 muscles [18] (i.e. semimembranosus, semitendinosus, biceps femoris long and short head, sartorius, tensor fasciae latae, gracilis, soleus, tibialis posterior, tibialis anterior, flexor digitorum, flexor hallucis, peroneus brevis, peroneus longus, peroneus tertius and extensor digitorum) were identified by non-rigidly registering the musculoskeletal model's generic tibial geometry to the participants' surface mesh via the coherent point drift algorithm [27]. Muscle forces were applied as a concentrated nodal force at the identified insertion points in the direction of the muscle's line of action. The ankle joint forces and muscle forces applied to the tibias can be found in electronic supplementary material, S1.

#### 2.1.4. Outcome variables

FE simulations were solved using FEBio (v. 3.2) [28]. From the simulations, we extracted effective strain from the periosteal surface of the tibial shaft—defined as 15–75% of tibia length. This differs from previous research which used a definition of 20–80% of tibia length [14]; however, visual inspection revealed 15–75% was a better representation of the tibial shaft for the dataset used in our study. As in previous work, elements within 1 cm of the soleus and 0.5 cm of all other muscles were ignored when strain was extracted due to artefacts causing unnaturally high strains [14].

### 2.2. Statistical analysis

We used the non-parametric permutation method described by Pataky *et al*. [29] to conduct classical hypothesis testing at the whole model (i.e. tibial shaft) level. Specifically, we used this approach with linear regression models to evaluate the relationship between geometric characteristics (i.e. dependent variable) and effective strain along the tibial shaft (i.e. independent variable). All statistical analysis was performed in MATLAB (R2021, Mathworks, MA, USA).

The dependent variables for the model were select principal component (PC) scores from three previously created statistical shape models of the: (i) outer cortical surface of the tibia, (ii) combined outer cortical surface of the tibia and fibula, and (iii) combined model of the cortical and trabecular surfaces [12]. All models and segmentations used in this study can be found at https://simtk.org/projects/ssm_tibia.

Prior to undertaking these analyses, we assessed collinearity across PC scores between the different shape models to avoid investigating related components (see electronic supplementary material S2). We used a Pearson's correlation coefficient cut-off between components of greater than 0.6 to identify related components. This allowed us to minimize collinearity (i.e. via an observed reduction in variance inflation factor) of the components included in our analyses while maintaining an adequate description of tibia-fibula geometric variation. When correlated components were identified (*r* > 0.6), only one of the correlated PC scores was selected for linear regression. Where components from the tibia-only shape model were correlated with components from the tibia-fibula shape model, those from the tibia-fibula model were prioritized for inclusion. We prioritized those from the tibia-fibula shape model due to the fibula having a potential role in bracing the tibia under loads [18]. Further, components that accounted for greater variation in a shape model were prioritized. Seven of the 17 PC scores across the three shape models were removed from statistical analyses, resulting in the final set of 10 dependent variables (table 1). The PC scores (i.e. dependent variables) were then input alongside the effective strain across the tibial shaft (i.e. independent variable) in separate linear regression models. The linear regression models generated a *t*-statistic across the elements of the tibial shaft—whereby a positive and negative *t*-value represented an increase and decrease in strain at the given element, respectively, with a concurrent increase in the PC score associated with the shape model. An alpha level of 0.05 was used to calculate the critical *t*-value which denoted statistical significance.

**Table 1.** PCs retained for statistical analysis.

shape model	PC	shape variation (%)	geometric variation observed with higher PC scores
tibia-fibula model	PC1	84.24	shorter and thinner tibia and fibula
	PC2	3.50	thicker fibula midshaft, with a more posterior head relative to the tibia
	PC3	2.60	overall thinner tibia and fibula
	PC4	1.91	straighter fibula with a more posterior head; the tibia displayed increased anterior–posterior curvature
	PC5	1.36	a more posteriorly curved fibula, and a more externally rotated tibial plateau
	PC6	1.03	thicker mid-upper tibia with increased condyle prominence
	PC7	0.85	increased interosseous width at the distal end of the tibia and fibula
cortical- trabecular model	PC2	4.46	an increase in midshaft cortical thickness, with decreased diameter of the medullary cavity
	PC3	1.46	increased anterior curvature of both the cortical and trabecular bone
	PC4	1.02	increase in cortical thickness and a decreased trabecular bone volume at the ends of the tibia

## 3. Results

**Figure 1.**
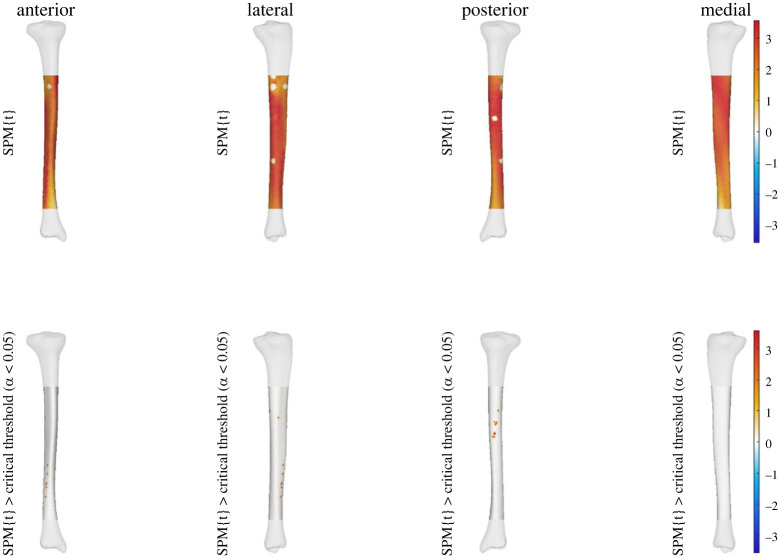
Linear regression outputs for PC6 of the tibia-fibula shape model at 3 m s^−1^. A higher score for PC6 describes increased thickness of the midshaft to upper tibia with an increase in condyle size. The top row shows the relationship between effective strain and increases in PC6 scores. The bottom row shows where these were when this relationship reached statistical significance. Warmer colours indicate a relationship between higher PC scores and increases in effective strain, while cooler colours indicate decreases in effective strain. Small areas of no strain represent muscle attachment sites that were masked from analysis.

**Figure 2.**
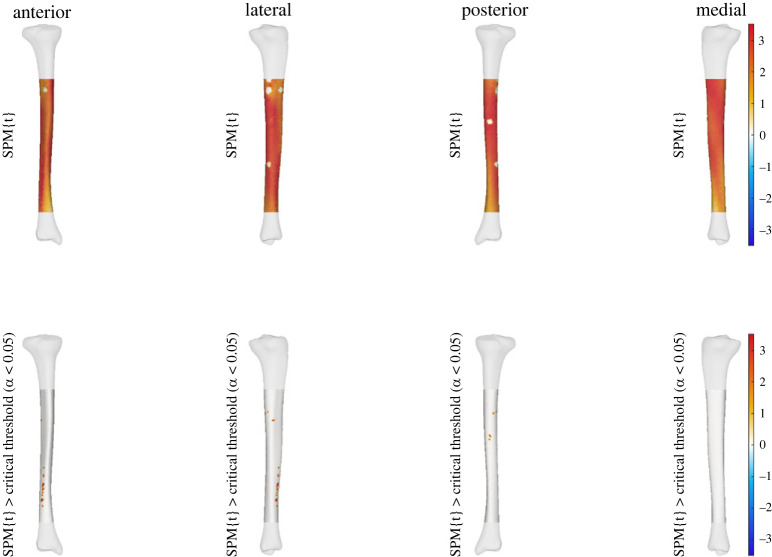
Linear regression outputs for PC6 of the tibia-fibula shape model at 4 m s^−1^*.* A higher score for PC6 describes increased thickness of the midshaft to upper tibia with an increase in condyle size. The top row shows the relationship between effective strain and increases in PC6 scores. The bottom row shows where these were when this relationship reached statistical significance. Warmer colours indicate a relationship between higher PC scores and increases in effective strain, while cooler colours indicate decreases in effective strain. Small areas of no strain represent muscle attachment sites that were masked from analysis.

**Figure 3.**
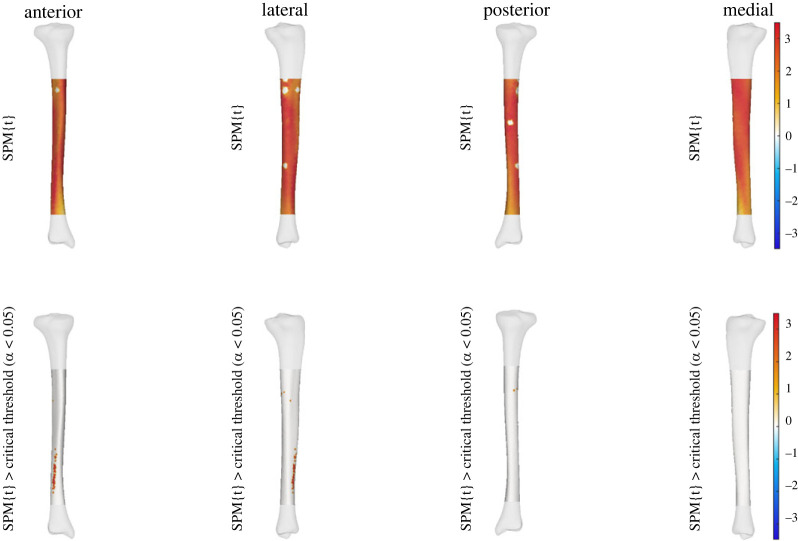
Linear regression outputs for PC6 of the tibia-fibula shape model at 5 m.s^−1^*.* A higher score for PC6 describes increased thickness of the midshaft to upper tibia with an increase in condyle size. The top row shows the relationship between effective strain and increases in PC6 scores. The bottom row shows where these were when this relationship reached statistical significance. Warmer colours indicate a relationship between higher PC scores and increases in effective strain, while cooler colours indicate decreases in effective strain. Small areas of no strain represent muscle attachment sites that were masked from analysis.

We observed a statistically significant relationship between the thickness of the midshaft to upper tibia and condyle prominence (i.e. PC6 of tibia-fibula model) to effective strain across areas of the tibial shaft. A higher score for this shape characteristic (i.e. increased thickness of the midshaft to upper tibia with increase in condyle prominence) was related to increases in effective strain at specific points along the distal anterolateral and proximal posterior regions of the tibial shaft at all three running speeds. This relationship between PC6 and effective strain was also seen across the entire tibial shaft at all speeds; however, it was not statistically significant (see figures 1–3).

**Figure 4.**
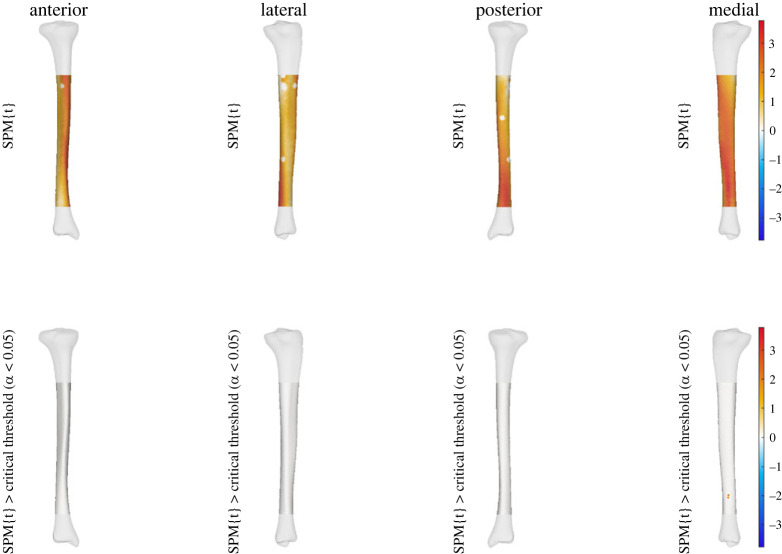
Linear regression outputs for PC2 of the cortical-trabecular shape model at 5 m s^−1^. A higher score for PC2 describes increased midshaft cortical thickness with a decrease in medulla cavity diameter. The top row shows the relationship between effective strain and increases in PC2 scores. The bottom row shows where these were when this relationship reached statistical significance. Warmer colours indicate a relationship between higher PC scores and increases in effective strain, while cooler colours indicate decreases in effective strain. Small areas of no strain represent muscle attachment sites that were masked from analysis.

We also observed a statistically significant relationship between medullary cavity and midshaft cortical thickness (i.e. PC2 of cortical-trabecular model) to effective strain across areas of the tibial shaft. A higher score for this shape characteristic (i.e. increased midshaft cortical thickness and decreased width of the medullary cavity) was related to increases in effective strain at specific points along the medial aspect at the distal end of the tibial shaft at running speeds of 5 m s^−1^. Similar to previous findings, the relationship between PC2 and effective strain was also seen across the entire tibial shaft; however, it was not statistically significant (figure 4).

There was no statistically significant relationship between the remaining PCs (i.e. PC1, PC2, PC3, PC4, PC5 and PC7 from the tibia-fibula shape model; PC3 and PC4 from the tibia cortical-trabecular model) and effective strain. Results and descriptions for linear regression models across all shape model components can be found in the electronic supplementary material, document S3.

## 4. Discussion

This study investigated the effect of tibia-fibula variation on tibial strain magnitude when running loads at various speeds were applied to participant-specific bone models. An increase in thickness of the midshaft to upper tibia with an increase in condyle prominence was related to increased effective strain at various points of the distal anterior–lateral border of the tibial shaft. The direction of this relationship was also present across the entire tibial shaft but did not reach statistical significance. This relationship was identified across all running speeds examined. Further, an increase in midshaft cortical thickness and a decrease in medulla cavity diameter were associated with increased effective strain at points of the distal medial tibial shaft when loads from running at 5 m s^−1^ were applied. Further, the direction of this relationship was observed in the area surrounding the statistically significant points; however, it did not reach statistical significance. This suggests that although the statistically significant areas are small, the geometric variations are associated with some level of increased strain magnitude in the surrounding areas.

Tibias with an overall increase in thickness, including thicker cortical bone, are associated with increased bone strength and an increased ability to withstand loading [30]. However, we found an inverse relationship between tibial strain in the distal third to increases in width isolated to the mid to upper section of the tibia (i.e. elevated strain with increased width). Although this was only found to be significant in small areas, we did see the same trend at surrounding areas on the bone. It is plausible that this occurs due to the relative thickness of the upper shaft compared with the lower shaft. The shape model characteristic in our study described an increase in tibia width in the upper portion, with no changes in the lower portion, causing the distal tibia shaft to be relatively thinner. This potentially causes redistribution of strain to areas more susceptible to the applied loads (i.e. the distal end). Contrary to our hypothesis, we did not observe a relationship between strain and bone thickness when variations to overall tibial width (i.e. PC3 of the tibia-fibula model) and overall size (including both size and width; i.e. PC1 of the tibia-fibula model) were explored. Recent work has proposed that tibial size has the biggest impact on tibial strain during running, where Bruce *et al*. [14] found increases in tibial size were related to reduced strain. A major difference between our study and previous work [14] is that the loads applied were scaled to participant's body mass, whereas Bruce *et al*. [14] kept loads consistent irrespective of tibial size. We demonstrated that within our study sample, height and body mass explained 63–64% of variance in tibial size [12], suggesting that the majority of variation in tibial size and thickness is relative to height and body mass [31]. Although shorter and lighter individuals have smaller thinner tibias, they may produce reduced forces and moments during running that can be adequately resisted. When loads are relative to body mass, changes in overall width and size of the tibia may have little effect on strain across the tibia. When considered in conjunction with existing work, our findings suggest that an individual with an imbalance between the thickness of the upper and distal portions of the tibial shaft, or a smaller overall tibia relative to their height and mass may be at risk of elevated tibial strain during running.

Increased curvature of long bones increases bending and decreases strength when the bone is subjected to axial compression [32,33]. Increases in tibial shaft curvature could modify the moment arm of applied loads, contributing to further bending and a potential increase in strain in the tibial shaft. Our results did not support this theory, with no statistically significant relationship found between changes in anterior–posterior bend of the tibial shaft (PC5 of the tibia-fibula model, PC3 of the trabecular-cortical model) and effective strain. This also contradicts recent findings from Bruce *et al*. [14], who found models with shorter tibias and fibulas with increased sagittal plane curvature had an increase in tibial strain of between 5.7% and 11.5%. Recent work has proposed the fibula acts as a structural brace reducing the magnitude of tibial bending [18]. The shape characteristic in our model that described increased anterior–posterior curvature of the tibia also described a straighter fibula*.* We propose that this corresponding straighter fibula present in our study sample increased its bracing capabilities and subsequently compensated for the increased curvature of the tibia—resulting in no strain-related association with this shape characteristic.

Bone morphology of long bones has been identified to change over time, particularly, to withstand the internal loading environment [34,35]. Dependent on how bone has remodelled over time, it is likely that participants have different geometric variations that could impact the strain response when different loads are applied. The running loads used in this paper were taken from previous literature and hence are generic in nature. Individuals probably display different running technique, and joint and muscle force profiles during running—and this should be considered alongside our findings. The generic loads used in the strain estimates may not accurately recreate the internal loading environment for the specific individual. The variability in geometries may simply be a consequence of normal bone development and when participant-specific loads for habitual activities are applied may be able to withstand these loads. The geometric variations found to increase strain may only be hazardous to the individual when the tibia experiences strains that are non-habitual. The modifications to the internal loading environment could potentially see the tibia unable to withstand this new strain pattern. Future research should aim to use participant-specific running data alongside participant-specific models to create more realistic loading scenarios. Further, understanding changes in tibial geometry throughout growth and how this could impact strain magnitude and tibial stress fracture risk in later life should be a topic of future research.

## 5. Limitations

A limitation of our methodology was the inability to distinguish if other geometric variations were affecting the relationship between our independent variable (i.e. shape characteristics) and effective strain. It is possible that certain shape characteristics that increase tibial strain concurrently occur with shape characteristics that offset any potential increase. While this limitation can be addressed by developing ‘synthetic’ tibia-fibula models by isolating shape characteristics (i.e. as per Bruce *et al*. [14]), examining participant-specific models potentially provides a more realistic estimate of tibial geometric characteristics. Combined shape characteristics may have specific effects on tibial strain during running. Future research should therefore consider how the interactions between or combinations of shape characteristics alter tibial strain.

As in our previous work [12], the tibia models used in this research were predicted to be from healthy adults (using meta-data provided by the NMDID [15]). Most participants' meta-data included past medical history; however, we cannot confirm that participants had no prior illness or injury that affected bone growth and formation. The results estimated from these models may also not be applicable to adults outside the ages of 19–40, particularly paediatric and geriatric populations.

Finally, our study cannot directly link geometric variations of the tibia with tibial stress fractures. Currently there is little knowledge on the exact magnitude of strain that results in developing a stress fracture—and this probably differs from one individual to another. It is understood that strain magnitude and increases in normal strain magnitude can lead to increased microdamage, progressing towards a stress response [3]. Further there are other influencing factors such as previous damage accumulation, which make it difficult to determine a threshold [3]. It is therefore difficult to directly link the magnitude of tibial strain to fracture risk; we can only infer that increases could increase risk. Probabilistic stress fracture models would be the best way to assess this [6,19]. Future research should consider applying probabilistic stress fracture models in the context of changes in geometry and strain magnitude.

## 6. Conclusion

Increased upper-midshaft tibial thickness with increased condyle prominence, and increased midshaft cortical thickness were associated with increased effective strain in the tibial shaft when running loads were applied. Increased anterior–posterior bend of the tibial shaft and overall size of the tibial shaft were not related to changes in tibial strain. When applied running loads are relative to body mass, overall width and size of the tibia may have little effect on tibial strain. An individual may be at risk of elevated tibial strain when there is an imbalance between the thickness of the upper and distal portions of the tibial shaft, or they have a smaller overall tibia relative to their height and mass.

## Data Availability

The raw data and code for this project is available at https://github.com/aaronsfox/tibial-geometry-loading. The data for the previously created statistical shape model can be found here: https://simtk.org/projects/ssm_tibia. The CT scans obtained from the New Mexico Decedent Image Database cannot be shared publicly as registration and approval is required from the creators. However, the public can access these through the appropriate steps: https://nmdid.unm.edu/. Individuals wishing to view the scans will need to register and request the appropriate medical imagery. The case IDs for the scans can be found in the above-linked repository under the segmentation folder. When requesting the images, these can be used to acquire the correct scans. The data are provided in the electronic supplementary material [36].

## References

[1] Rizzone KH, Ackerman KE, Roos KG, Dompier TP, Kerr ZY. 2017 The epidemiology of stress fractures in collegiate student-athletes, 2004–2005 through 2013–2014 academic years. J. Athl. Train. **52**, 966-975. (10.4085/1062-6050-52.8.01) 28937802 PMC5687241

[2] Taunton JE. 2002 A retrospective case-control analysis of 2002 running injuries. Br. J. Sports Med. **36**, 95-101. (10.1136/bjsm.36.2.95) 11916889 PMC1724490

[3] Pattin CA, Caler WE, Carter DR. 1996 Cyclic mechanical property degradation during fatigue loading of cortical bone. J. Biomech. **29**, 69-79. (10.1016/0021-9290(94)00156-1) 8839019

[4] Warden SJ, Burr DB, Brukner PD. 2006 Stress fractures: pathophysiology, epidemiology, and risk factors. Curr. Osteoporos Rep. **4**, 103-109. (10.1007/s11914-996-0029-y) 16907999

[5] Burr DB, Milgrom C, Boyd R, Higgins RG, Radin EL. 1991 Experimental stress fractures of the tibia. Clin. J. Sport Med. **1**, 70. (10.1097/00042752-199101000-00018) 2341429

[6] Edwards WB, Taylor D, Rudolphi TJ, Gillette JC, Derrick TR. 2009 Effects of stride length and running mileage on a probabilistic stress fracture model. Med. Sci. Sports Exerc. **41**, 2177-2184. (10.1249/MSS.0b013e3181a984c4) 19915501

[7] Popp KL, Frye AC, Stovitz SD, Hughes JM. 2019 Bone geometry and lower extremity bone stress injuries in male runners. J. Sci. Med. Sport **23**, 145-150. (10.1016/j.jsams.2019.09.009) 31594711

[8] Beck BR, Rudolph K, Matheson GO, Bergman AG, Norling TL. 2014 Risk factors for tibial stress injuries. Clin. J. Sport Med. **25**, 230-236. (10.1097/jsm.0000000000000126) 24977954

[9] Crossley KM, Bennell KL, Wrigley T, Oakes BW. 1999 Ground reaction forces, bone characteristics, and tibial stress fracture in male runners. Med. Sci. Sports Exerc. **31**, 1088-1093. (10.1097/00005768-199908000-00002) 10449008

[10] Cosman F, Ruffing J, Zion M, Uhorchak J, Ralston S, Tendy S, McGuigan FEA, Lindsay R, Nieves J. 2013 Determinants of stress fracture risk in United States Military Academy cadets. Bone **55**, 359-366. (10.1016/j.bone.2013.04.011) 23624291

[11] Bruce OL, Baggaley M, Welte L, Rainbow MJ, Edwards WB. 2021 A statistical shape model of the tibia-fibula complex: sexual dimorphism and effects of age on reconstruction accuracy from anatomical landmarks. Comput. Methods Biomech. Biomed. Engin. **25**, 875-886. (10.1080/10255842.2021.1985111) 34730046

[12] Keast M, Bonacci J, Fox A. 2023 Geometric variation of the human tibia-fibula: a public dataset of tibia-fibula surface meshes and statistical shape model. PeerJ **11**, e14708. (10.7717/peerj.14708) 36811007 PMC9939022

[13] Quintens L, Herteleer M, Vancleef S, Carette Y, Duflou J, Nijs S, vander Sloten J, Hoekstra H. 2019 Anatomical variation of the tibia – a principal component analysis. Sci. Rep. **9**, 1-10. (10.1038/s41598-019-44092-8) 31114000 PMC6529455

[14] Bruce OL, Baggaley M, Khassetarash A, Haider IT, Edwards WB. 2022 Tibial-fibular geometry and density variations associated with elevated bone strain and sex disparities in young active adults. Bone **161**, 116443. (10.1016/j.bone.2022.116443) 35589067

[15] Edgar H, Daneshvari Berry S, Moes E, Adolphi N, Bridges P, Nolte K. 2020 *New* *Mexico Decedent Image Database*. Albuquerque, NM: Office of the Medical Investigator, University of New Mexico. (10.25827/5s8c-n515)

[16] Moerman KM. 2018 GIBBON: the geometry and image-based bioengineering add-on. J. Open Sour. Softw. **3**, 506. (10.21105/joss.00506)

[17] Si H. 2015 TetGen, a Delaunay-based quality tetrahedral mesh generator. ACM Trans. Math. Softw. **41**, 1-36. (10.1145/2629697)

[18] Haider IT, Baggaley M, Edwards WB. 2020 Subject-specific finite element models of the tibia with realistic boundary conditions predict bending deformations consistent with in vivo measurement. J. Biomech. Eng. **142**, 021010. (10.1115/1.4044034) 31201743

[19] Edwards WB, Taylor D, Rudolphi TJ, Gillette JC, Derrick TR. 2010 Effects of running speed on a probabilistic stress fracture model. Clin. Biomech. (Bristol, Avon) **25**, 372-377. (10.1016/j.clinbiomech.2010.01.001) 20096977

[20] Rho JY. 1996 An ultrasonic method for measuring the elastic properties of human tibial cortical and cancellous bone. Ultrasonics **34**, 777-783. (10.1016/S0041-624X(96)00078-9) 9010460

[21] Renault JB, Aüllo-Rasser G, Donnez M, Parratte S, Chabrand P. 2018 Articular-surface-based automatic anatomical coordinate systems for the knee bones. J. Biomech. **80**, 171-178. (10.1016/J.JBIOMECH.2018.08.028) 30213649

[22] Marchetti DC, Moatshe G, Phelps BM, Dahl KD, Ferrari MB, Chahla J, Turnbull TL, LaPrade RF. 2017 The proximal tibiofibular joint: a biomechanical analysis of the anterior and posterior ligamentous complexes. Am. J. Sports Med. **45**, 1888-1892. (10.1177/0363546517697288) 28339288

[23] Beumer A, van Hemert WLW, Swierstra BA, Jasper LE, Belkoff SM. 2003 A biomechanical evaluation of the tibiofibular and tibiotalar ligaments of the ankle. Foot Ankle Int. **24**, 426-429. (10.1177/107110070302400509) 12801200

[24] Hamner SR, Delp SL. 2013 Muscle contributions to fore-aft and vertical body mass center accelerations over a range of running speeds. J. Biomech. **46**, 780-787. (10.1016/j.jbiomech.2012.11.024) 23246045 PMC3979434

[25] Seth A, Sherman M, Reinbolt JA, Delp SL. 2011 OpenSim: a musculoskeletal modeling and simulation framework for *in silico* investigations and exchange. Procedia IUTAM **2**, 212-232. (10.1016/j.piutam.2011.04.021) 25893160 PMC4397580

[26] Van Arkel RJ, Modenese L, Phillips ATM, Jeffers JRT. 2013 Hip abduction can prevent posterior edge loading of hip replacements. J. Orthop. Res. **31**, 1172-1179. (10.1002/JOR.22364) 23575923 PMC3736148

[27] Myronenko A, Song X. 2010 Point set registration: coherent point drifts. IEEE Trans. Pattern Anal. Mach. Intell. **32**, 2262-2275. (10.1109/TPAMI.2010.46) 20975122

[28] Maas SA, Ellis BJ, Ateshian GA, Weiss JA. 2012 FEBio: finite elements for biomechanics. J. Biomech. Eng. **134**, 011005. (10.1115/1.4005694) 22482660 PMC3705975

[29] Pataky TC, Koseki M, Cox PG. 2016 Probabilistic biomechanical finite element simulations: whole-model classical hypothesis testing based on upcrossing geometry. PeerJ Comput. Sci. **2**, e96. (10.7717/peerj-cs.96)

[30] Ammann P, Rizzoli R. 2003 Bone strength and its determinants. Osteoporos. Int. **14**, 13-18. (10.1007/s00198-002-1345-4) 12730800

[31] Duyar I, Pelin C. 2003 Body height estimation based on tibia length in different stature groups. Am. J. Phys. Anthropol. **122**, 23-27. (10.1002/AJPA.10257) 12923901

[32] Bertram JE, Biewener AA. 1988 Bone curvature: sacrificing strength for load predictability? J. Theor. Biol. **131**, 75-92. (10.1016/s0022-5193(88)80122-x) 3419194

[33] Jade S, Tamvada KH, Strait DS, Grosse IR. 2014 Finite element analysis of a femur to deconstruct the paradox of bone curvature. J. Theor. Biol. **341**, 53-63. (10.1016/J.JTBI.2013.09.012) 24099719

[34] Frost HM. 1964 Bone biodynamics. Boston, MA: Little Brown and Co.

[35] Wolff J, Maquet P, Furlong R. 1986 The law of bone remodelling. Berlin, Germany: Springer-Verlag. See https://books.google.com.au/books?id=RwJPAQAAIAAJ.

[36] Keast M, Bonacci J, Fox A. 2023 Variability in tibia-fibular geometry is associated with increased tibial strain from running loads. Figshare. (10.6084/m9.figshare.c.6845561) PMC10523080 37771963

